# Ultrasound-Guided Percutaneous Bone Biopsy: Feasibility, Diagnostic Yield and Technical Notes

**DOI:** 10.3390/diagnostics13101773

**Published:** 2023-05-17

**Authors:** Federico Ponti, Alessio Arioli, Chiara Longo, Marco Miceli, Marco Colangeli, Nicolas Papalexis, Paolo Spinnato

**Affiliations:** 1 Diagnostic and Interventional Radiology, IRCCS Istituto Ortopedico Rizzoli, 40136 Bologna, Italypaolo.spinnato1982@gmail.com (P.S.); 2 Orthopaedic Oncology Unit, IRCCS Istituto Ortopedico Rizzoli, 40136 Bologna, Italy

**Keywords:** bone neoplasms, image-guided biopsy, radiology, interventional, ultrasonography

## Abstract

While nowadays, CT-guided bone biopsy represents the gold standard tool for histopathological and microbiological diagnosis of skeletal lesions, the role of US-guided bone biopsy has not yet been fully explored. US-guided biopsy offers several advantages, such as the absence of ionizing radiation, fast acquisition time, as well as good intra-lesional echo, and structural and vascular characterization. Despite that, a consensus in regard to its applications in bone neoplasms has not been established. Indeed CT-guided technique (or fluoroscopic ones) still represents the standard choice in clinical practice. This review article aims to review the literature data about US-guided bone biopsy, underlying clinical-radiological indications, advantages of the procedure and future perspectives. Bone lesions taking the best advantages of the US-guided biopsy are osteolytic, determining the erosion of the overlying bone cortex and/or with an extraosseous soft-tissue component. Indeed, osteolytic lesions with extra-skeletal soft-tissue involvement represent a clear indication for US-guided biopsy. Moreover, even lytic bone lesions with cortical thinning and/or cortical disruption, especially located in the extremities or pelvis, can be safely sampled with US guidance with very good diagnostic yield. US-guided bone biopsy is proven to be fast, effective and safe. Additionally, it offers real-time needle evaluation, an advantage when compared to CT-guided bone biopsy. In the current clinical settings, it seems relevant to select the exact eligibility criteria for this imaging guidance since the effectiveness can vary depending on the type of lesion and body site involved.

## 1. Introduction

Nowadays, CT-guided biopsy represents the most used imaging-guidance technique to characterize bone lesions since it provides high-resolution images of the needle and of the targeted area, showing great diagnostic accuracy [1,2,3].

Ultrasound-guided (US-guided) biopsy is routinary used for histopathological diagnosis of a variety of organs but has always occupied a secondary role in the field of bone lesions, mainly because of the signal attenuation caused by the compact bone.

The US-guided procedure shows many advantages, compared to CT-guided and fluoroscopic-guided ones, such as the absence of radiation exposure, lower cost, fast acquisition-time, real-time needle evaluation, and vascular assessment by the power-color-Doppler tool [4]. US-guided biopsy has been exploited for just a small set of lesions, especially osteolytic ones, or with wide cortical disruption.

Lately, various technologies have been explored to overcome Ultrasound limits, such as integrative tools of needle tracking and MRI/CT fusion imaging [5].

The aim of our article is to review the literature regarding US-guided bone biopsy, describing technical notes of the procedures, pointing out safety, effectiveness, advantages and disadvantages. Moreover, we aim to summarize the current possible clinico-radiological indications for US-guided biopsy of skeletal lesions.

### 1.1. Percutaneous Bone Biopsy: General Indications

Percutaneous bone biopsy is currently the recommended first-line method for histopathologic and/or microbiologic identification of bone lesions. The approach of choice for evaluating skeletal lesions suspected of being cancerous (suspicious primary bone tumors or systemic cancer metastases) involves this instrument.

There are many clinical indications to perform an imaging-guided skeletal biopsy. Confirming metastases in a patient with a known underlying malignancy is one of the most frequent indications [6]. Even while anamnestic data and radiological examinations support the diagnosis of bone metastases, image-guided biopsy is recommended not just for histopathologic confirmation [6,7,8]. In fact, deeper molecular and receptor characterization of metastatic bone lesions is possible, with implications for patient care and treatment selection.

Some well-known indications for bone biopsy include recurrence detection of bone tumors and tumor therapy response (for example, the percentage of necrosis found following therapies). A bone biopsy is also advised in cases of unexpected non-traumatic fractures. A known indication for these procedures is specifically establishing the nature of a non-traumatic vertebral collapse [9].

Another indication is to investigate suspected bone infections (microbiological analysis) or to make a differential diagnosis between an infection, particularly spondylodiscitis, and other bone lesions, as well as soft-tissue infections [9,10]. The typical method for diagnosing osteomyelitis (or spondylodiscitis) involves combining clinical, laboratory and imaging data [11,12]. When blood cultures reveal pathogenic bacteria, percutaneous biopsy is typically advised for the patient.

It is crucial to be aware that surgical incisional biopsy enables the collection of bigger samples in the case of suspected primary bone sarcomas of the appendicular skeleton in order to perform further molecular and personalized therapeutic tools [13]. This means that if a radiographic characteristic of primary bone sarcoma is evident, the patient may be submitted right away for an incisional surgical biopsy, avoiding percutaneous techniques in these selected cases. In different clinical settings, the choice of surgical biopsy as the first approach on the suspect of sarcoma may vary, depending on institutional experience and lesion site.

### 1.2. Imaging Guidance for Percutaneous Bone Biopsy

With the guidance of fluoroscopy, CT, or ultrasound, a skeletal biopsy can be safely carried out. The most used imaging technology for bone biopsy right now is CT guidance, which makes it possible to safely sample practically all the skeletal locations. There are some peculiar and risky skeletal sites in which CT guidance is the only recommended tool, above all the skull, the cranio-vertebral junction and cervical spine [14,15,16,17,18,19,20].

There is still a place for fluoroscopy-guided skeletal biopsy, usually in clinical settings where CT guidance is not available. Orthopedic surgeons typically perform the fluoroscopic technique in surgical rooms. However, the Ultrasound guidance could be safely used instead of the fluoroscopic one in many cases. The lengthy bones of the extremities, where the accuracy of CT imaging guidance is not strictly required, are a good candidate for this option.

Indeed, US-guided biopsy is a very intriguing tool, which provides the advantages of real-time analysis and precise assessment of neighboring soft tissues, especially with regard to neurovascular bundles [21]. In regard to this latter point, an Ultrasound with the aid of power-color-Doppler is an optimal tool to assess in real-time the position of vascular bundles, allowing the operator to make a real-time evaluation of the targeted area, safely avoiding vessels from the needle’s trajectory.

In Table 1, we summarized the main radiological and anatomical indications for the clinical application of US-guided bone biopsy.

## 2. Technical Notes and Overview of Approaches

Fine Needle Aspiration (FNA) and Core Needle Biopsy (CNB) can both be performed under Ultrasound guidance to assess bone lesions.

The linear probe is the preferred transducer within authors using a range of frequencies between 4.5 and 7.5 MHz. Depending on the depth of the lesion, the curved probe (convex) can be employed using 3–4 MHz for deeply located bone lesions [21,22]. Special Ultrasound transducers with biopsy guides and channel brackets are also available. It would be beneficial to standardize the choice of the probes in the different skeletal sites, whatever the difference in patients’ body characteristics.

Depending on the type of sample needed, the size of the needle can vary. FNA is usually performed with a small needle, from 20 to 25 Gauge (G), while wider calipers are reserved for CNB procedures, often ranging between 11 and 18 G.

The majority of authors still prefer conducting the biopsy “Free-hands” (meaning without any auxiliary accessories) even though recently, many guiding accessories have been developed, such as needle tracking (also known as coaxial technique) and fusion imaging (merging the Ultrasound real-time evaluation with previously acquired CT or MRI studies). The latter consents to perform the real-time US biopsy under the guidance of previous MRI/CT scans and represent an innovative way to overcome the visualization limits in those lesions without cortical disruption [5]. Pre-operative management seems to be quite homogeneous, comprising clotting profile testing within the previous 48 h (according to SIR and CIRSE guidelines) with, when required, suspension of anticoagulants/antiplatelet agents (depending on clinical settings).

Consultation of previous imaging exams (CT/MRI) is mandatory to identify the lesion characteristics as well as its anatomical relationship with adjacent important structures, such as vessels and nerves, and to meticulously plan the operative time.

Ultimately, an additional ultrasound evaluation is required to confirm the procedure feasibility. The procedure starts with disinfection and sterile draping. Local anesthesia is usually administered from skin to periosteum using lidocaine, mepivacaine, or levobupivacaine. A small incision of the skin facilitates the trocar’s introduction (especially for larger needles). Under ultrasound guidance, the trocar is advanced firstly until cortical contact and then gradually until the region of interest.

Once the needle is placed, the inner stylet is removed, and the biopsy can be performed manually. If correctly collected, the bone sample can be sent for histopathological or microbiological analysis, and the small wound is usually eased with a sterile bend or steri-strips.

After the procedure, patients receive one to two hours of clinical surveillance.

Pictorial Exemplification Cases of US-Guided Bone Biopsy

In this subsection, we provided a series of cases from our Institution’s experience in regard to US-guided bone biopsy, in order to provide graphical exemplification of the clinical application of this tool in musculoskeletal oncology.

**Figure 1:**
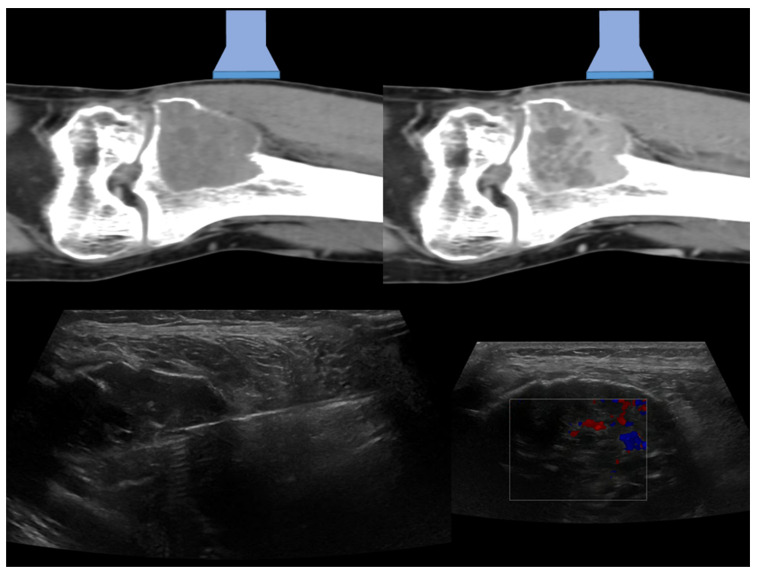
In the upper part of the figure are represented CT coronal images before (on the **left**) and after (on the **right**) contrast media injections, with a graphical representation of the ultrasound probe position (linear probe). In the lower right corner, the coronal ultrasound images of the lesion with Color-Doppler evaluation that allowed the detection of the vascularization of the lesion and to guide the needle biopsy (left–14 G TruCut needle) procedure is represented. A subsequent histological diagnosis of a giant cell tumor was achieved.

Case 1: A 27-years-old male with osteolytic lesion of the left tibial metaepiphysis (Figure 1).

**Figure 2:**
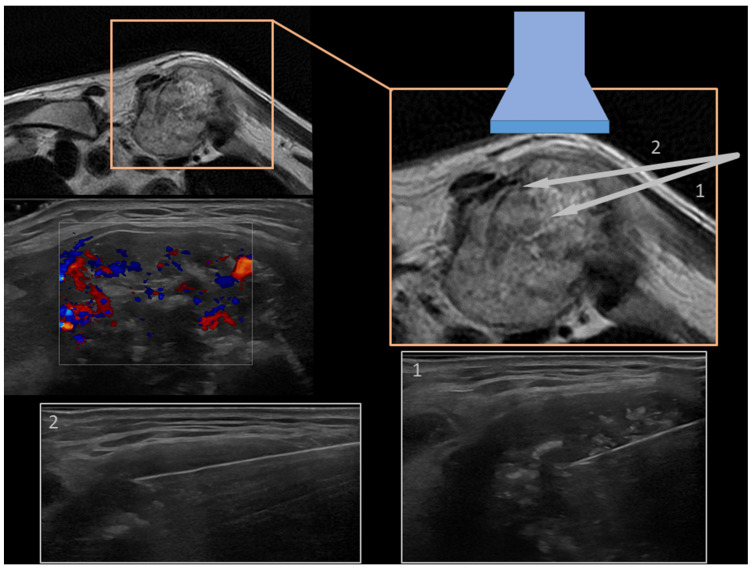
On the upper-left part of the figure, MRI axial image and the ultrasound Color-Doppler evaluation, showing a strong internal vascularization, is shown. The magnification on the right (orange square) highlights graphically the schematic position of the probe and the approach of the needle (14 G–TruCut). In panel 1 and panel 2 are depicted the US-guided biopsies with the real-time visualization of the needle. The final histopathological diagnosis was chordoma metastasis.

Case 2: A 79-years-old male with an osteolytic lesion of the left clavicle with extra-osseous component (Figure 2).

**Figure 3:**
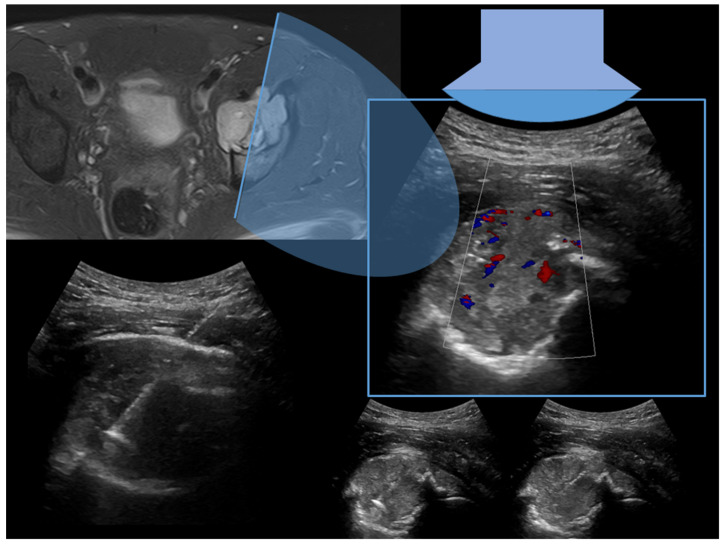
MRI axial T2-weighted sequence with fat suppression demonstrated an osteolytic-aggressive lesion of left acetabulum infiltrating iliopsoas muscle (34-years-old man) with complete cortical disruption. On the right is depicted a sagittal ultrasound scan (box with sky-blue border) using a convex probe. The lower part of the figure depicts the approach of the needle (11 G coaxial trap-system) in the lesion. A final histopathological diagnosis of undifferentiated cancer metastasis was obtained.

Case 3: A 34-year-old male with osteolytic and painful lesion of the left acetabulum (Figure 3).

**Figure 4:**
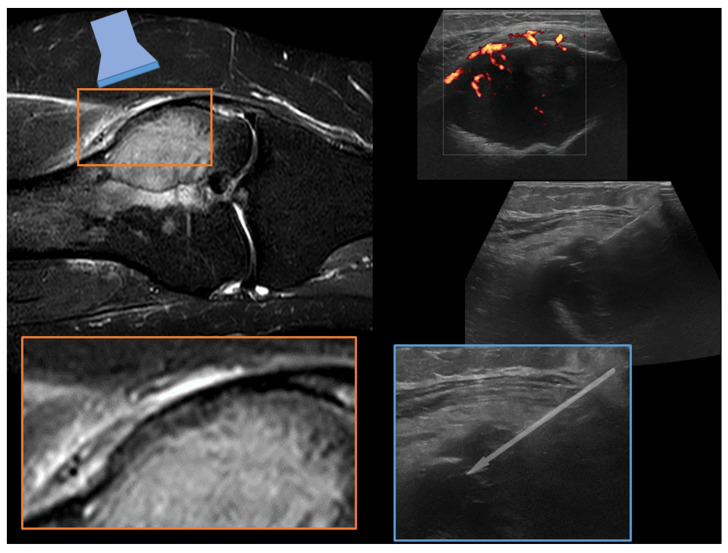
MRI T2-weighted fat-suppressed coronal sequence (**upper-left**) shows a lesion of the proximal femur without cortical disruption. The magnification (**lower-left** orange square) emphasizes a thinned cortex (on the **left**) which allows an adequate visualization of the lesion at the ultrasound assessment (on the upper-right part of the figure). The needle real-time assessment during the biopsy procedure is visualized on the lower-right part of the figure (sky-blue square) where the path of the needle towards the most vascularized area of the lesion is shown (gray arrow). A final histopathological diagnosis of giant cell tumor of bone was obtained with the biopsy sample.

Case 4. A 51-year-old woman with osteolytic lesion of the lateral femoral condyle (Figure 4).

## 3. Success Rate and Feasibility

Only a few brief reports or series with weak or scant evidence have examined the US-guided biopsy technique, which is specifically used for bone lesions. The diagnostic yield (i.e., the percentage of procedures leading to a specific pathological diagnosis) or accuracy (i.e., the percentage of procedures leading to the proper diagnosis) of US-guided bone biopsies were frequently reported in studies, but sensibility or specificity were usually left out.

In the literature, the accuracy and the diagnostic yield of US-guided bone biopsies are estimated to be between 86.2% and 100% and between 78% and 100%, respectively, mostly depending on lesion and biopsy type, target location, and the operator that performed the biopsy [5,21,22,23,24,25,26,27,28,29,30,31,32,33,34,35,36,37,38,39,40,41,42,43,44,45,46,47,48,49].

### 3.1. Type of Lesion

The US-guiding technique can be useful in assessing several types of bone lesions. According to Gil-Sanchez et al., bone lesions can be clustered into four types (1) lytic with extraosseous components, (2) lytic with disrupted bone cortex, (3) sclerotic and (4) without modification of the bone cortex. The first two types of bone lesion (osteolytic) are most likely to receive the diagnostic biopsy under US guidance since the ultrasonic beam reaches the lesion obtaining a greater visualization and allowing the assessment of vascularization with the Color or Power-Doppler technique, reaching a high diagnostic yield. On the other hand, independent of the guiding technique (US or CT), sclerotic lesions were linked to a lower diagnostic yield [36].

In types 1 and 2 bone lesions, the biopsy was generally performed with cutting or fine needles, while trephine needles (Jamshidi, Ostycut, Bonopty) were used for the latter types of bone lesions, allowing drilling in the bone cortex and cutting biopsy or aspiration with a coaxial technique [36].

### 3.2. Type of Biopsy

#### 3.2.1. Type of the Needle

In order to collect an appropriate tissue sample, the kind of needle used can be crucial. In earlier studies [24,25,26,27,28,33], FNA was preferred, while CNB is preferred in more recent research [43,45]. The accuracy of FNA and CNB were only compared in one study, and the results showed that CNB had a better diagnostic yield in type 1 lesions, where a statistically significant difference was obtained [36]. The width of the needle has not been sufficiently assessed until today. The diagnostic yield of the various biopsy needle gauges (14-15-16-18 G) was only examined in one research [41], and no statistically significant difference between the needle sizes was discovered. This study had two limitations: the use of thinner needles and the greater number of CT- (133 patients) vs. US- (18 patients) guided biopsies.

#### 3.2.2. Needle Approaches and Cores

The quantity of obtained cores and the amount of needle passages are additional crucial factors. Three needle passages were typically sufficient to result in the highest diagnostic yield [41]. The studies that investigated this matter found that the sample size had a significant impact on diagnostic yield. Longer samples (<5 mm—42%, 5–10 mm—61%, >10 mm—82%) were related to higher diagnostic yield [41].

#### 3.2.3. Characteristics of the Lesion

The success rate of the biopsy is influenced by the size of the bone lesion. On the accuracy of CT and US-guided biopsies, Wu et al. reported miscellaneous data [41]. With statistically significant variations, imaging-guided biopsies were the most effective at detecting the largest lesions: for diameters of less than 2 cm—54%, between 2 and 5 cm—75%, and greater than 5 cm—86% [41]. Since the authors did not only record accuracy for US-guided biopsies, there were some restrictions. Global accuracy ranged from 87.5 to 100% in studies that only reported the results of US-guided BL biopsies [23,24,25,26,32], but many of these studies [25,29] only analyzed a limited number of lesions. The diagnostic yield was still between 78 and 100% in trials with more than 15 lesions [26,27,28,32,33,34,35,40]. The capacity to discern between benign and malignant lesions and provide an accurate diagnosis of malignancy are the two most crucial diagnostic problems that can be resolved by any form of biopsy. In distinguishing benign from malignant lesions, US-guided biopsy showed excellent specificity (up to 100%) and very high sensitivity (95–96%). Data from the literature also showed that US-guided biopsy performed highly effectively (83–98%) in identifying specific types of malignancy. Furthermore, it is common knowledge that benign bone lesions tend to provide a lower diagnostic yield in all kinds of percutaneous bone biopsy than malignant ones. One such instance is osteoid osteoma, a benign, painful bone tumor with a histopathological diagnostic rate in percutaneous needle biopsy that is frequently less than 50% [50].

#### 3.2.4. Target Lesion Location and Hybrid Techniques

Some authors suggest using fusion-imaging technology to facilitate the biopsy in order to reach deep lesions or overcome beam attenuation in sclerotic lesions (in types 3 and 4 of the bone lesion). It blends real-time US imaging with the lesion’s prior CT or MRI scan. The research by Khali et al. [51], which combined US-guided biopsy with CT or MRI data, showed a diagnostic yield and accuracy similar to CT guidance but with quicker scheduling and biopsy times and lower treatment costs [51]. The authors examined 60 musculoskeletal samples, of which 17 were BL, but they omitted to specify the guiding technique applied in those cases.

Fourteen spine bone lesions (lumbar vertebral body, intervertebral disc and sacrum) without cortical disruption or intervertebral disc infection have been investigated by Mauri et al. For the first six cases, both standard CT and CT plus US fusion guidance were used. (Group 1). In the last eight cases, CT was only used after the needle had been inserted to ensure proper positioning under fusion imaging guidance. (Group 2). The radiologist reached the target lesion and acquired a bioptic sample in every instance, and both groups’ technical success and diagnostic yield were 100% [47]. The needle was positioned in the last eight operations using only fusion imaging guidance, and CT only served to verify its correct position after the needle had been placed. (Group 2). Groups 1 and 2 reported 100% technical success (the radiologist targeted the specified lesion and acquired a bioptic sample in every instance), and in both groups, a 100% diagnostic yield was observed [47].

In group 1 compared to group 2, the mean procedure time and median CT pass number were considerably longer (45 ± 5 vs. 26 ± 3 min and 7 vs. 3) [47].

Most of the possible benefits of fusion imaging have been considered for percutaneous liver biopsies; this technique has the potential to find and target challenging lesions, including lesions that are not visible on conventional ultrasound. Nevertheless, bone injuries can also be addressed by this technique. It appears to be feasible to use combined fusion-imaging and needle-tracking ultrasound guiding to target bone lesions without cortical interruption. Garnon et al. illustrated this approach in 2017. Despite the small number of patients included in their research, the outcomes were considerable: a mean treatment length time of 44 min with a diagnostic yield of 100% [5]. However, we believe that special attention must be paid when the fusion-imaging technique is applied for guiding biopsy in locations close to vital structures, such as vertebral bodies.

Contrast agents (Sonovue^®^) were occasionally used for US guiding of the bone lesions biopsies. To optimize the accuracy of musculoskeletal biopsy, a pilot study was developed to investigate the use of contrast agents. However, this research did not only evaluate bone biopsies; it also included a few bone lesions and demonstrated a 100% accuracy for the 25 musculoskeletal biopsies [52].

#### 3.2.5. Complications

Complications of the image-guided percutaneous bone biopsy are rare (less than 1%) [21,48].

The recognition that the majority of studies on US-guided bone biopsy did not record any complications is likely attributable to a number of factors, including the choice of lytic bone lesions for the biopsy, the target’s clear visibility, and the utilization of color Doppler mode (real-time vessels visualization).

Moreover, in the studies focused on US-guided ribs biopsy, to the best of our knowledge, the most considerable rate of complications reported were two cases (2/45 patients—4.4%) of post-procedure pneumothorax after rib biopsies that did not require drainage nor hospital admission [46].

## 4. Discussion

Ultrasonography is routinely employed worldwide as a guiding technique for bone biopsy, showing an accuracy rate and sensibility of 72–99%, quite comparable data to the standard CT-guided procedure (94%) [1,21]. The main reason ultrasound guidance is highly accepted by patients and operators is because it is a cheap, fast, radiation-free tool with the possibility of bedside application [21,53].

It is a real-time, multiplanar, guiding imaging technique that offers instantaneous visualization of the target lesion and of the bioptic needle, and it presents many diagnostic futures facilitating the guided procedure, such as the Doppler (Power/Color) tool, helpful in the identification of neurovascular structures, which increases both safety and diagnostic yield, while decreasing the complication rate.

The literature reports just a small number of biopsies executed with the US-guide compared to those executed under CT guidance, and the quality of evidence is relatively low since fundamental statistical parameters, such as sensibility or specificity, are not commonly reported.

Furthermore, musculoskeletal ultrasound is one of the most operator-dependent imaging techniques since the lower experience of the performer physician is often related to a lower procedure success rate. From a technical point of view, the bone cortex highly attenuates the ultrasonic beam reducing the spatial resolution of the methodic.

Further studies are needed to evaluate the relationship between the effectiveness of the US-guided biopsy and the thickness of the cortical bone overlying the lesion.

Additionally, pathologic lesions of superficial bones can be eligible to receive US-guided biopsy, as shown in Table 1; the procedure, in fact, reached a 97.8% accuracy both with FNA and CNB, as investigated by Huang et al. on rib lesions. Since CT is characterized by a better spatial and contrast resolution in deep joints or vertebral bones, these body sites can benefit from the fusion technique already employed with promising results.

Image-guided bone biopsy complications are extremely uncommon, and there have only been a few occasions when US guidance was used (with the exception of US-guided rib biopsy–see above). Due to a number of factors, including the lytic type of bone lesion in US-guided biopsy, adequate target visibility and the potential for using color Doppler mode, the majority of studies did not report any problems.

US-guided bone biopsy is an intriguing tool that may present many advantages compared to CT-guided samples. Indeed, each bone lesion should be accurately evaluated, and the best imaging guidance option should be chosen in order to improve patients’ care. The main advantages and disadvantages of US-guided bone biopsy are summarized in Table 2.

To establish an eventual improved function of US-guided bone lesions biopsy for bone pathology, more studies involving US-guided biopsies with uniform data reporting are needed. To reach an agreement among experts on the procedure’s time and bone lesion eligibility, additional studies must be carried out.

The procedure needs to be further investigated to establish an expert consensus on lesion eligibility and the intra-operative time.

## 5. Conclusions

US-guided biopsy of bone is an interesting tool that needs further investigation to assess its role in the management of bone lesions in musculoskeletal oncology.

Several advantages render this tool the optimal choice, particularly in some radiological patterns and some skeletal locations. The application of fusion imaging to this tool, merging the real-time Ultrasound with previous CT or MRI studies, renders its use even more interesting and precise. Indeed, the use of combined fusion imaging and US-guided real-time needle assessment can be applied to target bone lesions even without cortical disruption or soft-tissue extensions.

Further investigations focusing on the use of US guidance for the biopsy of bone lesions are encouraged.

## Figures and Tables

**Table 1:** Summary of possible clinical applications/indications of US-guided bone biopsy.

Affected Skeletal Region	Radiologic Pattern	Ultrasound Guidance
Appendicular skeleton	Sclerotic pattern, osteolytic or mixed pattern without cortical disruption or intramedullary only involvement	+/−
Appendicular Skeleton	Osteolytic or mixed pattern with cortical disruption or cortical thinning	++
Pelvic bones	Sclerotic lesions without large extra-skeletal soft-tissue involvement	+/−
Pelvic bones	Osteolytic or mixed with large soft-tissue extra-skeletal involvement	+
Spine	All patterns (intraosseous lesions)	−
Spine	Large lesions of vertebral body (even if with extra-osseous component)	−
Spine	Lesions with posterior large extra-skeletal component	++
Ribs	Osteolytic or mixed pattern with cortical disruption or cortical thinning	++
Ribs	Sclerotic pattern, osteolytic or mixed pattern without cortical disruption or intramedullary only involvement	+/−
Sternum	All	+/−
Sternum	Anterior extra-skeletal soft-tissue involvement	++
Skull and Craniovertebral junction	All	−

Table Legends: ++ = the use is suggested, + = the use is possible, +/− = the use can be applied only in selected cases, − = the use is discouraged.

**Table 2:** Summary of advantages and disadvantages of US-Guided bone biopsy compared to CT-Guided ones.

Advantages of US-Guided Bone Biopsy	Disadvantages of US-Guided Bone Biopsy
Fast Acquisition-Time	Absence of visualization of intramedullary bone if cortex is preserved
Less expansive compared to CT-guided ones	Not safely applicable in all skeletal sites (e.g., deep one, vertebral body)
Real-time needle evaluation	Operator experience dependent
Evaluation of vascular structures (extra- and intra-lesion)	
Precision of target areas of lesions (avoiding necrosis or fluid collection)	
Absence of ionizing radiation exposure	

## Data Availability

Not applicable.
